# How Do Shifts in Patients with Mental Health Problems’ Formal and Informal Care Utilization Affect Informal Caregivers?: A COVID-19 Case Study

**DOI:** 10.3390/ijerph192416425

**Published:** 2022-12-07

**Authors:** Leonarda G. M. Bremmers, Leona Hakkaart-van Roijen, Eleonora S. Gräler, Carin A. Uyl-de Groot, Isabelle N. Fabbricotti

**Affiliations:** Erasmus School of Health Policy and Management, Erasmus University Rotterdam, P.O. Box 1738, 3000 DR Rotterdam, The Netherlands

**Keywords:** informal caregiver, substitution of formal care, COVID-19 pandemic

## Abstract

(1) Background: This study investigated how potential shifts in patients’ formal and informal care utilization during the COVID-19 pandemic impacted their informal caregivers in terms of their subjective burden, psychological wellbeing, and happiness. (2) Methods: A retrospective cohort study design was employed for a panel of Dutch informal caregivers of persons with mental health problems (*n* = 219) in June 2020. Descriptive statistics and differences between means were determined for the patients’ informal and care utilization and informal caregivers’ subjective burden, happiness, and psychological wellbeing. Three mediation analyses were conducted using the PROCESS macro. (3) Results: Informal caregivers reported significantly worse happiness and subjective burden scores during the COVID-19 pandemic compared with before the lockdown. There were minimal shifts in patient’s care utilization reported, with the exception of a decrease in significant emotional and practical support provided by the informal caregiver. In the mediation analyses, there was not a significant indirect effect of shifts in patients’ formal care utilization on informal caregivers’ subjective burden, psychological wellbeing, and happiness through shifts in patients’ informal care utilization. (4) Discussion and conclusion: Whilst we found that shifts in patients’ care utilization during the first wave of the pandemic did not affect the informal caregiver in the short term, it is unclear what the long-term impact of the pandemic might be on informal caregivers. More research should be conducted to understand the implications of short- and long-term impact of substitution on informal caregivers of persons with mental health problems, with special consideration of the COVID-19 context and uptake of e-health technology.

## 1. Introduction

Over the past decades, psychiatric care has undergone fundamental changes in the Netherlands with the locus of care shifting from institutions to the community [1]. These reforms have been characterized by the integration of ambulatory services, implementation of community-based mental health centres, de-hospitalization of patients, and differentiation within sheltered housing accommodation [1,2]. The successful implementation of these reforms relies on the support networks of patients. Consequently, the public and personal domains of psychiatric care have blurred, with patients’ loved ones assuming an increased amount of the patients’ care [3,4], hereinafter referred to as informal care. Informal care is defined as the “long-term care or support lent on voluntarily basis to a family member, friend, or acquaintance for their mental health problems” [5] (p. 6). Given the increasing burden of mental disorders, personnel shortages, ageing population, and increasing healthcare costs, further long-term mental health policy and system reforms are expected to be implemented across Europe [1,6,7,8,9]. These reforms emphasize a further reliance on informal care to compensate for cutbacks in professional and residential home care [7].

Informal care provision has a lasting and widespread impact on the lives of informal caregivers [10]. For example, informal caregivers report significantly lower levels of wellbeing compared with non-caregivers [11]. Compared with informal caregivers for patients with somatic disorders, informal caregivers for patients with psychiatric disorders report a higher perceived burden of care, hereinafter referred to as subjective burden [12]. Hence, informal caregivers for persons with mental health problems are considered a more vulnerable group of caregivers that require additional support and attention [13]. The nature and complexity of mental disorders, inadequate support from health care professionals, and disorder-related stigma all contribute to a worse caregiving experience [14]. Concerningly, high subjective burden scores have been associated with adverse health outcomes [15,16], poor quality of life ratings [17,18], and higher mortality rates [16]. Moreover, highly burdened informal caregivers, such as informal caregivers of persons with mental health problems [12], also have lower psychological wellbeing [19,20] and happiness scores [21].

**Figure 1:**
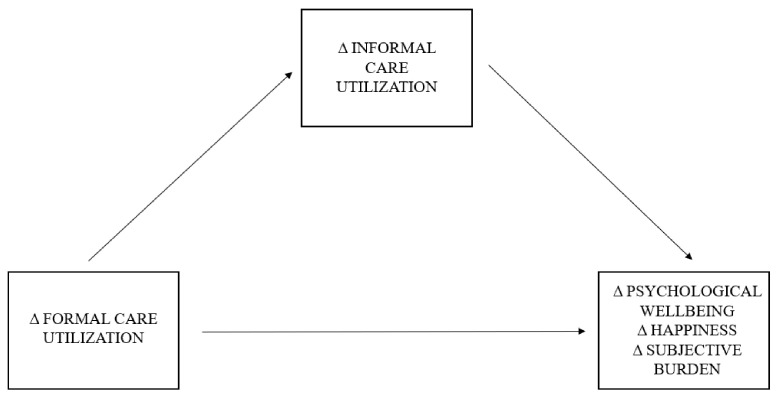
Conceptual diagram of proposed mediation model. *Notes:* Δ = difference between two time points.

As the reliance on informal caregivers is expected to increase over the coming years [22], we must consider how these respective shifts in patients’ care utilization may impact the informal caregiver. The relationship between informal and formal care has been addressed by several theories [23,24,25]; nevertheless, these theories do not consider how substitution of patient’s formal care impacts their informal caregiver. Empirical research has demonstrated that as professional long-term care resources continue to decrease, the wellbeing gap for informal caregivers continues to increase [11], suggesting a direct relationship between changes in patient’s formal care utilization and informal caregiver outcomes. These shifts in formal care utilization, however, also affect the provision of informal care, with a 1% decrease in formal care associated with a 4–6% increase in informal care hours for patients with schizophrenia and bipolar disorder [26]. Similar results have been found for elderly care, where an increase in formal care has been associated with a significant decrease in the probability informal care use [27]. These findings indicate that shifts in patients’ care utilization can result in long-term substitution [26,27] and a causal effect of changes in patient’s formal care utilization on their informal care utilization. Furthermore, an increase in care intensity (i.e., hours of informal care provision) have been linked to role overload [28], worse psychological wellbeing [29], and a higher subjective burden [30,31] in informal caregivers. These findings indicate that shifts in patient’s informal care utilization is a causal antecedent of informal caregiver outcomes. Hence, shifts in patient’s informal care may serve as a mediator and facilitate the relationship between shifts in patient’s formal care utilization and informal caregiver outcomes, such as subjective burden, psychological wellbeing, and happiness (see Figure 1).

The first wave of the COVID-19 pandemic provides a unique opportunity to investigate the potential impact of shifts in patients’ care utilization, given that mental health services were amongst the most severely disrupted [32]. The COVID-19 pandemic resulted in the (partial) suspension and/or closure of health and social support services and an increased reliance on informal caregivers [33]. The limited empirical research on the subject matter indicate that caregiving burden was adversely affected by the COVID-19 pandemic and lockdown restrictions. Research conducted at the start of the lockdown found that a majority of informal caregivers in the Netherlands reported an increase in subjective burden [34]. These results were confirmed in an additional Dutch caregiver panel study, which reported that the (partial) suspension of non-emergency health and social support services resulted in an increased subjective burden compared with before the COVID-19 pandemic. When compared with non-caregivers, informal caregivers also had poorer mental and physical health [35]. COVID-19-specific stressors may have contributed to these adverse outcomes, such as care recipients being at hospital while one is restricted to visit them and care recipients being at increased risk for a serious course of the disease in case of an infection [36]. Despite the severe disruption of mental health services during the first wave of the COVID-19 pandemic, no research has been conducted to investigate whether shifts in patients’ informal and formal care utilization occurred and whether these shifts influence informal caregivers in terms of subjective burden, happiness, and psychological wellbeing.

### Research Aim and Questions

Hence, this study aims to shed light on the potential shifts in informal and formal care utilization of persons with mental health problems and the subsequent consequences for their informal caregivers, using the first wave of the COVID-19 pandemic as a case study. The following sub-questions were formulated to address the research aim:

1. How were patients’ formal and informal care utilization and informal caregivers’ subjective burden, happiness, and psychological wellbeing affected by the COVID-19 pandemic?
2. To what extent did shifts in patients’ formal and informal care utilization affect informal caregivers’ subjective burden, happiness, and psychological wellbeing?
3. To what extent did shifts in patients’ informal care utilization mediate the relationship between shifts in patients’ formal care utilization and informal caregivers’ subjective burden, happiness, and psychological wellbeing?

## 2. Materials and Methods

### 2.1. Study Design and Participants

A retrospective cohort study design was employed, wherein an online questionnaire was administered to a panel of Dutch informal caregivers (*n* = 1, 006) in June 2020, which was 3 months after the start of the lockdown in the Netherlands [37]. People were urged to social distance, remain at home when possible, and minimize unnecessary travelling. Healthcare and social services were partially suspended at this time. The lockdown had a score of 63 on the Oxford Stringency Index for the period of data collection [38].

Respondents were asked to assess their caregiving situations—current (T1) and before the lockdown (T0) with a recall period of 3 months. Respondents were considered eligible for inclusion if they reported that they were adults (≥18 years of age) and provided a minimum of 2 h of informal care per week to an adult (≥18 years of age) with a known psychological disorder or psychosocial problem for at least a 3-month period. For the final analysis, 219 respondents were included. A total of 730 respondents were excluded because they did not comply with the inclusion criteria; hence, 57 respondents were excluded due to missing or unreliable responses.

### 2.2. Ethical Approval

The study protocol was reviewed and approved by the Research Ethics Review Committee of the Erasmus School of Health Policy & Management (reference: IRB 20–16). All respondents provided informed consent prior to completing the survey and were free to stop participation at any moment in time.

### 2.3. Measurements

Data were collected using questionnaires that assessed the socio-demographic characteristics of the informal caregiver and their care recipient, the caregiving situation, the care recipients’ utilization of formal and informal care (T0, T1), subjective burden (T0, T1), happiness (T0, T1), and psychological wellbeing (T0, T1).

#### 2.3.1. Socio-Demographic Characteristics

Socio-demographic information of the informal caregiver included child caregiving responsibility, care recipient comorbidity status, caregiver age and gender, employment status, and care recipient living situation. Care recipient comorbidities included temporary or long-term physical disability, terminal illness, dementia or other memory-related disorders, intellectual disability, and age-related complaints. We also administered self-developed questions which asked informal caregivers about their COVID-19-related concerns (no concerns/some concerns/very concerned/not applicable) regarding the following statements “that they will get infected with COVID-19 due to their contact with their care recipient”, “that their care recipient will get infected with COVID-19 due to their contact with the caregiver”, “loneliness of their care recipient”, and “that they will not be able to care for their care recipient”.

#### 2.3.2. Independent Variables

Informal care use was assessed using an adapted version of the Intensity of Informal Care Questionnaire [5]. Emotional support was added to the original set of informal care tasks, support with household tasks, support with self-care tasks, and practical support. The care recipients’ weekly utilization of these informal care tasks was assessed for T0 and T1. The unit of measurement was hours per week.

To determine formal care use, respondents were asked to indicate whether their care recipients’ use of formal care services changed during the COVID-19 pandemic (no change in formal care utilization/decrease in formal care utilization/increase in formal care utilization) for the following services: home help, meals received from a catering service, physical care and support from a homecare organization, nursing home admittance, daytime activities, social work, appointments with the psychologist, psychiatrist or psychotherapist, group therapy, outpatient care, mental health clinic admittance, and psychiatric hospital admittance. The iMTA Medical Consumption Questionnaire [39] was used to identify relevant health services.

#### 2.3.3. Dependent Variables

Subjective burden was assessed using the self-rated burden scale (SRB), which employs a horizontal visual analogue scale (VAS) to judge the burden of caregiving on a scale from 0 (“not straining at all”) to 10 (“much too straining”) [40]. The SRB has been validated as a generic measurement instrument and can be used in different informal caregiving populations and research settings. It can also be used as a screening tool to detect severe burden amongst informal caregivers [41].

The valuation component of the Care-related Quality of Life instrument (CarerQol), a horizontal VAS measuring wellbeing of the informal caregiver in terms of general happiness, was employed to determine happiness. The CarerQol VAS ranges from 0 (“completely unhappy”) to 10 (“completely happy”) [42]. The CarerQoL has demonstrated favorable psychometric properties for informal caregivers of long-term care users [43].

Psychological wellbeing was assessed with the Mental Health Quality of Life (MHQoL)-VAS. The MHQoL is a standardized, self-administered quality of life measure that was developed for use in people with mental health problems. The scale ranges from 0 (“worst imaginable psychological well-being”) and 10 (“best imaginable psychological well-being”). The MHQoL has demonstrated good psychometric properties for the general population and patients in treatment for their mental health problems [44].

### 2.4. Procedure

Changes in total informal care use were calculated by subtracting the sum score of hours of informal care given at T0 from the sum score of hours at T1. Changes in total formal care use were coded: 0 = “no change”, 1 = “increase in utilization”, and 2 = “decrease in utilization”. Categorical covariates were dichotomized: care recipient living situation (0 = “does not live with informal carer”, 1 = “lives with informal carer”), caregiver employment status (0 = “unemployed”, 1 = “full- or part-time employment”), care recipient comorbidity status (0 = “no, does not have a comorbidity”, 1 = “yes, has at least one comorbidity”), child caregiving responsibility (0 = “not caring for a child under the age of 18”, 1 = “caring for a child under the age of 18”), and caregiver gender (0 = “male”, 1 = “female”). COVID-19-related concerns were aggregated and coded as 0 = “no concerns”, 1 = “some concerns”, and 3 = “very concerned”.

### 2.5. Statistical Analyses

Statistical analyses were conducted in Stata/MP 17.0 and IBM SPSS Statistics 27 with a statistical significance (α) of 0.05 to analyse the research questions. The complete data analysis syntax is available upon request.

Descriptive statistics (mean, standard deviation (*SD*), and frequency distributions) were computed to report the characteristics of caregivers, care recipients, and the caregiving situation.

#### 2.5.1. Sub-Question 1: How Did Patients’ Formal and Informal Care Utilization and Informal Caregivers’ Subjective Burden, Happiness, and Psychological Wellbeing Change during the COVID-19 Pandemic?

The mean and *SD* were determined for the patients’ informal care utilization (hours per week) and informal caregivers’ subjective burden, happiness, and psychological wellbeing at T1, T0, and the difference between T0 and T1 (Δ: T0-T1). To assess the differences in informal care utilization before and during the COVID-19 pandemic, a Wilcoxon signed-rank test was administered. Due to the nominal nature of the formal care utilization variable, additional descriptive statistical analyses (frequency distributions) were completed for all forms of care utilization. Paired t-tests were administered to assess the differences in means (T0, T1) for informal caregivers’ subjective burden, happiness, and psychological wellbeing.

#### 2.5.2. Sub-Question 2: How Did Shifts in Patients’ Formal and Informal Care Utilization Affect Informal Caregivers’ Subjective Burden, Happiness, and Psychological Wellbeing?

Caregivers were categorized into the following nine groups on the basis of the reported shift in their care recipients’ formal and informal care utilization during the COVID-19 pandemic: no change in formal care hours, decrease in informal care hours (*n* = 11); increase in formal care hours, decrease in informal care hours (*n* = 4); decrease in formal care hours, decrease in informal care hours (*n* = 8); no change in formal care hours, no change in informal care hours (*n* = 93); increase in formal care hours, no change in informal care hours (*n* = 16); decrease in formal care hours, no change in informal care hours (*n* = 29); no change in formal care hours, increase in informal care hours (*n* = 31); increase in formal care hours, increase in informal care hours (*n* = 15); and decrease in formal care hours, increase in informal care hours (*n* = 12). For each group, the mean and *SD* of the scores on the informal caregivers’ subjective burden, happiness, and psychological wellbeing at T0 and T1 were calculated. The within-group differences were then assessed for each group with a paired *t*-test.

#### 2.5.3. Sub-Question 3: To What Extent Did Shifts in Patients’ Informal Care Utilization Mediate the Relationship between Shifts in Patients’ Formal Care Utilization and Informal Caregivers’ Subjective Burden, Happiness, and Psychological Wellbeing?

A mediation analysis was conducted for the dependent variables (Y): the change in informal caregivers’ subjective burden, happiness, and psychological wellbeing. For the mediation analyses, shifts in patients’ total formal care utilization served as the independent variable (X) and the shifts in patients’ total informal care utilization the mediator (M). Due to the multi-categorical nature of the dependent variable, two dummy variables were generated with “no change in formal care utilization” as the base (X1:1 = “increase in formal care hours”, 0 = “no change in formal care hours”; X2:1 = “decrease in formal care hours”, 0 = “no change in formal care hours”).

All analyses were run using model 4 of the PROCESS macro (version 4.0) for SPSS with 10,000 bootstrap samples [45]. Each analysis included the following covariates: overall COVID-19-related concerns, child caregiving responsibility, care recipient comorbidity status, caregiver age, caregiver gender, caregiver employment status, and care recipient living situation. All continuous independent variables were centered.

## 3. Results

### 3.1. Characteristics of Respondents

Table 1 displays the socio-demographic characteristics of the informal caregivers and care recipients. The mean age of informal caregivers was 47.4 years (*SD* = 16.1 years) and 40.2% identified as male. A total of 60.7% of informal caregivers were engaged in full- or part-time employment. Only 19 informal caregivers did not have overall COVID-19-related concerns (8.9%). A majority of respondents did not care for a child under the age of 18 (70.3%). The care recipients largely lived independently or in an institution (72.6%). In addition to the existing psychosocial problems or psychiatric disorder, their caregiver reported that 67.1% of the care recipients had a comorbidity.

### 3.2. Reported Changes during The COVID-19 Pandemic: Patients’ Care Utilization and Informal Caregiver Outcomes (Sub-Question 1)

#### 3.2.1. Patients’ Care Utilization

During the COVID-19 pandemic, informal caregivers reported providing less hours on total informal care provision (Δ = −0.6, *SD* = 4.7); however, this decrease was not statistically significant (*p* = 0.08). However, a significant decrease in informal care delivery was found for practical (*p* = 0.05) and emotional support tasks (*p* = 0.04). For formal care utilization, a majority of informal caregivers reported that their care recipient did not have a change in overall formal care utilization (*n* = 135, 61.6%) during the COVID-19 pandemic. A total of 49 care recipients (22.4%) received less formal care and 35 received more formal care (16.0%). The overall shifts in patients’ formal and informal care utilization can be found in Table 2.

#### 3.2.2. Informal Caregiver Outcomes

Worse outcomes were observed across all informal caregiver outcomes during the COVID-19 pandemic (T1) compared with before (T0), with a high degree of variability (refer to Table 3). A significant increase in subjective burden was observed when comparing average scores before the COVID-19 pandemic with during (Δ = 0.4, *SD* = 1.7, *p* < 0.01). In terms of happiness, a significant decrease in happiness was present (Δ = −0.6, *SD* = 1.5, *p* < 0.001). For psychological wellbeing, a decrease was observed; however, this decrease was not statistically significant (Δ = −0.1, *SD* = 1.3, *p* = 0.11). For a complete overview of the informal caregiver outcomes, refer to Table 3.

### 3.3. Differences between Caregiver Groups Reporting Shifts in Their Patients’ Formal and Informal Care Utilization (Sub-Question 2)

#### 3.3.1. Subjective Burden

Overall, shifts in patients’ care utilization resulted in an increase in subjective burden during the COVID-19 pandemic compared with beforehand (see Table 4). Significant increases in subjective burden at T1 were present for “decrease in formal care hours, no change in informal care hours” (Δ = 0.9, *SD* = 2.0, *t* = 2.5, *p* < 0.05) and “increase in formal care hours, increase in informal care hours” (Δ = 1.3, *SD* = 1.5, *t* = 3.2, *p* < 0.01). There were reported decreases in caregiver groups that reported a “decrease in formal care hours, increase in informal care hours” and “no change formal care hours, increase in informal care hours”; however, these changes were not statistically significant.

#### 3.3.2. Happiness

Across all combinations of informal and formal care shifts, caregivers reported a decrease in happiness (see Table 4). Significant decreases were observed in caregivers whose patients had a “decrease in formal care hours, decrease in informal care hours” (Δ = −1.0, *SD* = 0.8, *t* = −3.7, *p* < 0.01), “no change in formal care hours, no change in informal care hours” (Δ = −0.4, *SD* = 1.4, *t* = −2.6, *p* < 0.05), “decrease in formal care hours, no change in informal care hours” (Δ = −1.3, *SD* = 1.6, *t* = −4.3, *p* < 0.001), “no change in formal care hours, increase in informal care hours” (Δ = −0.5, *SD* = 1.3, *t* = −2.3, *p* < 0.05), and “increase in formal care hours, increase in informal care hours” (Δ = −1.3, *SD* = 1.8, *t* = −2.7, *p* < 0.05).

#### 3.3.3. Psychological Wellbeing

Shifts in patients’ informal and formal care utilization did not have an impact on informal caregiver’s wellbeing (see Table 4) despite the reported changes being mixed, with increases (*n* = 1), decreases (*n* = 5), and no change (*n* = 3) during the COVID-19 pandemic.

### 3.4. Effect of Shifts in Patients’ Formal Care Utilization on Informal Caregivers and Mediation by Shifts in Patients’ Informal Care Utilization (Sub-Question 3)

The complete mediation models can be found in the Supplementary Materials.

#### 3.4.1. Change in Subjective Burden

**Figure 2:**
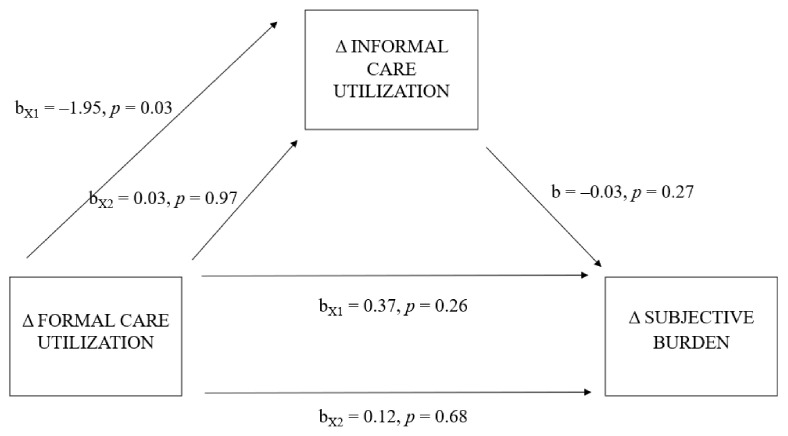
Model of shifts in formal care utilization as a predictor of change in subjective burden, mediated by shifts in informal care utilization. *Note.* Δ = difference between T0 and T1.

There was not a significant indirect effect of shifts in patients’ formal care utilization on informal caregivers’ subjective burden through shifts in patients’ informal care utilization (X1: b = 0.06, 95% bias-corrected and accelerated bootstrap confidence interval (BCa CI) [−0.05, 0.20]; X2: b = −0.001, 95% BCa CI [−0.06, 0.05]). The total effect model was insignificant (R2 = 0.07, F-value (9, 204) = 1.64, *p* = 0.17). The models predicting the indirect effect (a and b) were insignificant, R2 = 0.06, F-value (9, 204) = 1.54, *p* = 0.14 and R2 = 0.07, F-value (10, 203) = 1.60, *p* = 0.11, respectively. The model predicting the direct effect (c’) was also insignificant, R2 = 0.07, F-value (10, 203) = 1.60, *p* = 0.11. For the main regression coefficients, refer to Figure 2.

#### 3.4.2. Change in Happiness

**Figure 3:**
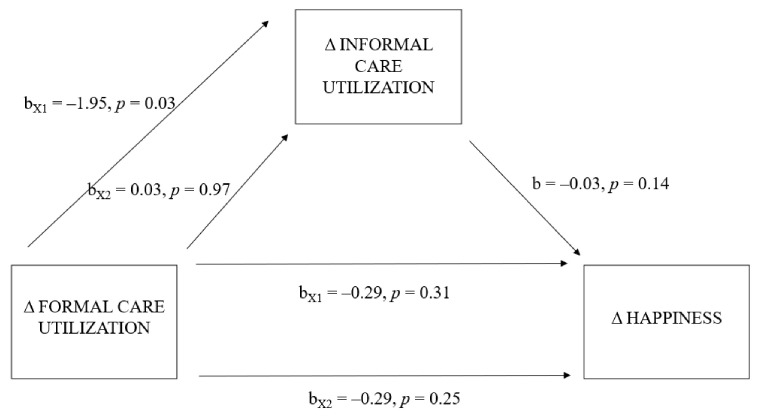
Model of shifts in formal care utilization as a predictor of change in happiness, mediated by shifts in informal care utilization. *Note.* Δ = difference between T0 and T1.

There was not a significant indirect effect of shifts in patients’ formal care utilization on informal caregivers’ happiness through shifts in patients’ informal care utilization (X1: b = 0.065, 95% BCa CI [−0.030–0.197]; X2: b = −0.001, 95% BCa CI [−0.055–0.061]). The total effect model was significant, R2 = 0.09, F-value (9, 204) = 2.32, *p* = 0.02. In the total effect model, overall COVID-19-related concerns (b = −0.449, 95% BCa CI [−0.781−0.118]) and child caregiving responsibility (b = 0.626, 95% BCa CI [0.142–1.111]) significantly predicted a change in informal caregiver’s happiness. The model predicting the indirect effect (a) was insignificant, R2 = 0.06, F-value (9, 204) = 1.54, *p* = 0.14, and the model predicting the indirect effect (b) was significant, R2 = 0.10, F-value (10, 203) = 2.32, *p* = 0.01. In the indirect effect model, overall COVID-19-related concerns (b = −0.463, 95% BCa CI [−0.794–−0.133]) and child caregiving responsibility (b = 0.597, 95% BCa CI [0.112–1.082]) significantly predicted a change in informal caregiver’s happiness (refer to the Supplementary Materials for the coefficients of the complete model). The model predicting the direct effect (c’) was also insignificant, R2 = 0.10, F-value (10, 203) = 2.32, *p* = 0.01. For the main regression coefficients, refer to Figure 3.

#### 3.4.3. Change in Psychological Wellbeing

**Figure 4:**
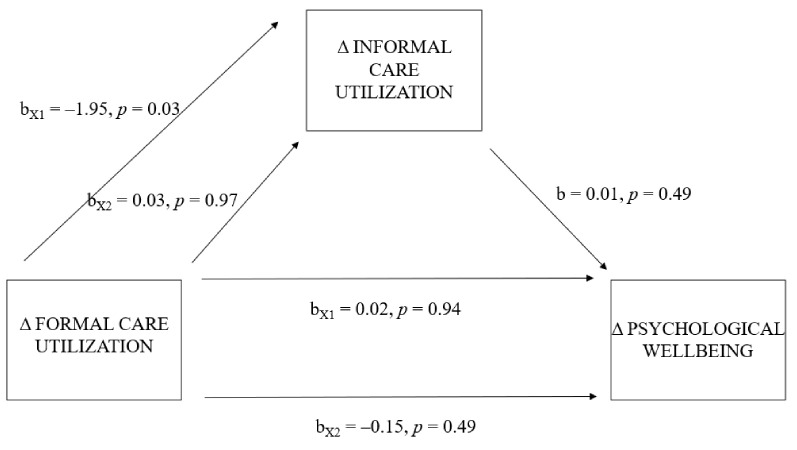
Model of shifts in formal care utilization as a predictor of change in psychological wellbeing, mediated by shifts in informal care utilization. *Note.* Δ = difference between T0 and T1.

There was not a significant indirect effect of shifts in patients’ formal care utilization on informal caregivers’ psychological wellbeing through shifts in patients’ informal care utilization (X1: b = −0.026, 95% BCa CI [−0.157–0.043]; X2: b = 0.001, 95% BCa CI [−0.046–0.024]). The total effect model was insignificant, R2 = 0.07, F-value (9, 204) = 1.56, *p* = 0.13. The models predicting the indirect effect (a and b) were insignificant, R2 = 0.06, F-value (9, 204) = 1.54, *p* = 0.14 and R2 = 0.07, F-value (10, 203) = 1.45, *p* = 0.16, respectively. The model predicting the direct effect (c’) was also insignificant, R2 = 0.07, F-value (10, 203) = 1.45, *p* = 0.16. For the main regression coefficients, refer to Figure 4.

## 4. Discussion

Family and other loved ones of persons with mental health problems continue to assume more caregiving responsibilities, as deinstitutionalization of the mental healthcare system has led to the substitution of formal care [7,22]. This trend is expected to continue; however, the impact on this specific group of informal caregivers is unknown. Hence, we investigated how potential shifts in patients’ formal and informal care utilization impacted their informal caregivers’ subjective burden, psychological wellbeing, and happiness, using the first wave of the COVID-19 pandemic as a case study.

We found that informal caregivers’ outcomes deteriorated at this time, with informal caregivers reporting significantly higher subjective burden and lower happiness scores compared with before the pandemic. With regard to their caregiving intensity, we found that compared with before the pandemic, the amount of informal care provided had decreased but only significantly with regard to emotional and practical support. In terms of care recipients’ formal care utilization, some care recipients received more care, some less care, but a majority of informal caregivers reported that their care recipient did not experience a change in formal care (61.6%) during the first wave of the pandemic. Upon further investigation, the shifts in patients’ formal and informal care utilization did not appear to have an impact on informal caregivers’ subjective burden, happiness, and psychological wellbeing. Moreover, patients’ informal care utilization did not mediate the relationship between shifts in patients’ formal care utilization and informal caregiver outcomes, and substitution of formal care did not occur. In conclusion, our study found that worse caregiver outcomes could not be accredited to short-term shifts in the patients’ formal or informal care utilization during the pandemic. Our findings contradict the existing literature on the impact of substitution and shifts in patients’ care utilization on informal caregivers.

### 4.1. Reflection on Main Findings

Findings from previous studies have established the detrimental impact that shifts in patients’ formal and informal care utilization can have on their informal caregivers [11,46,47,48,49,50]. The increased utilization of formal social support services, such as household and healthcare services, has been associated with lower levels of informal caregiver depression. Patient’s increased utilization of household and healthcare services offer their informal caregiver moments of respite and improve the patient’s health status, respectively [46]. Comparable results have been found for wellbeing outcomes [11]. Meanwhile, the increased provision of informal care has been associated with worse informal caregiver health outcomes and distress [47,48,49,50]. The increased provision of informal care can result in role conflict and role overload [28]. Role conflict is characterized by the incompatible expectations for the various informal caregiver roles that the informal caregiver needs to fulfill [51], whilst role overload occurs when informal caregivers have insufficient time and resources to complete their respective obligations [52]. The discrepancy between the literature and our study findings may be accredited to several factors, namely the minimal shifts in patients’ care utilization during the pandemic, no substitution of formal care, and the COVID-19 context.

#### 4.1.1. Minimal Shifts in Patients’ Care Utilization during the COVID-19 Pandemic

Overall, minimal shifts were observed in patients’ care utilization across our study findings and other studies conducted during the first wave compared with before the pandemic [53,54]. However, there are some discrepancies between our findings and other studies that investigated informal care provision during the first wave in the Netherlands [54,55,56]. Raiber and Verbakel also did not find that the amount of total informal care provision changed as a result of the COVID-19 pandemic [54], whilst other studies identified significant increases [55] and decreases [56] in informal care provision. These discrepancies may be accredited to the composition of the study population. Tur-Sinai et al. and Verbakel et al. focused on general informal care populations and did not have a particular focus on persons with mental health problems [55,56]. Uniquely, informal caregivers for persons with mental health problems often report feeling responsible for their loved ones and struggle with relinquishing their care responsibilities [57]. Moreover, persons with mental health problems have different care needs compared with other conditions [12].

Despite initial reports that mental health services were amongst the most severely disrupted during the pandemic [32], this was not reflected in patients’ utilization of formal care, as demonstrated in our study findings and the literature [53]. Chow et al. found that the number of mental health consultations remained within the normal pre-pandemic range of expected trend variation during the first wave, despite a significant drop in face-to-face out-patient contacts [53]. Face-to-face out-patient contacts were substituted with video consultations and telephone contacts [53]. An increased uptake of e-health tools was also reported for other healthcare services that are regularly employed by persons with mental health problems, such as general practitioner visits [58,59].

#### 4.1.2. No Substitution of Formal Care

When substitution of formal care occurs, it manifests as a negative relationship between the level of patients’ formal care utilization and informal care utilization, with informal caregivers increasing their care intensity in response to the insufficient formal care [25]. The substitution of formal care in response to healthcare reform has been demonstrated in several patient groups with long-term care needs [26,27,60,61,62]. Substitution of formal care may not have been observed in our study findings, due to the high proportion of emotional and practical support that was provided by our study population. Emotional and practical support are essential caregiving tasks provided by informal caregivers of persons with mental health problems [63,64] and are less susceptible to increased availability of formal care [27]. The provision of informal care originates from social bonds of reciprocity and existing relationships between the informal caregiver and care recipient [65], which allows informal caregivers to provide emotional support that cannot be substituted by formal care [23,27].

#### 4.1.3. COVID-19 Context

Due to patient’s formal and informal care utilization remaining relatively unchanged during the first wave, the COVID-19 pandemic may not have been an appropriate case study to study the impact of potential shifts in care utilization. As demonstrated in other works in the literature, the virus and respective lockdown measures had a detrimental impact that affected every aspect of society, including the economy, public health, and employment [66,67,68,69]. Particularly, vulnerable populations, such as people with mental health problems [69] and informal caregivers [33,35,70], were adversely affected. Thus, the impact of the pandemic may have been so widespread and intense that it offset any potential effects that patient’s care utilization shifts may have had. Furthermore, the time point of the COVID-19 pandemic should be considered due to the emergence of new viral variants. Numerous variants of the COVID-19 virus have emerged and caused “abrupt infectious waves” (p. 2618). This has required national governments to enact additional public health actions, such as further lockdown measures and epidemiological research [71].

### 4.2. Strengths and Limitations

This was a novel study that investigated how potential shifts in patients’ formal and informal care usage affected the wellbeing of their informal caregivers in the short term; however, there are some limitations that should be addressed. First, the study employed a retrospective cohort study design, which is susceptible to recall bias. This could result in the overestimation of the T0 measurements [72], meaning that it may have impeded our ability to observe differences between the T0 and T1 measurements of care utilization and informal caregiver outcomes. However, we expect that the likelihood of recall bias is limited due to the short recall period (3 months). Additionally, the observed shifts in care utilization and deterioration of informal caregiver outcomes were comparable to other Dutch studies that were also conducted around the same time. Second, all outcomes were caregiver-reported, including the formal care utilization of patients, and required the informal caregivers to have a good insight in their care recipients’ medical consumption. Previous studies have deemed proxy caregiver-reported outcomes a valid substitution for patient-reported outcomes [73]. Moreover, given that respondents were required to answer only a categorical question about changes in their care recipient’s formal care utilization, the risk of inaccuracy is low. Thus, we assume that the impact on this particular outcome is minimal.

## 5. Conclusions

Whilst we found that shifts in patients’ care utilization during the first wave of the pandemic did not affect the informal caregiver in the short term, it is unclear what the long-term impact of the pandemic might be on informal caregivers. The substitution of formal care did not occur, likely due to the high uptake of e-health solutions in the mental health sector during the first wave of the pandemic. Hence, informal caregivers were not required to increase their provision of care. Worse informal caregiver outcomes were observed during the pandemic, which could not be explained by shifts in patients’ care utilization. This is surprising, given that the relationship between shifts in patients’ care utilization and informal caregiver outcomes are well-established in other contexts, such as deinstitutionalization and de-centralization of mental health care. More research should be conducted to understand the short- and long-term impact of substitution on informal caregivers of persons with mental health problems, using qualitative and quantitative research methods. Future research should consider the role of context, such as the COVID-19 pandemic and increased uptake of e-health technology.

## Figures and Tables

**Table 1:** Socio-demographic Characteristics of Informal Caregivers and Care Recipients (*n* = 219).

Informal Caregivers	
Gender	
Male *n* (%)	88 (40.2)
Age *mean years (min.-max.; SD)*	47.4 (18–84; 16.1)
Employment status	
Full- or part-time employment *n* (%)	133 (60.7)
Overall COVID-19-related concerns	
No concerns *n* (%)	19 (8.9)
Some concerns *n* (%)	77 (36.0)
Very concerned *n* (%)	118 (55.1)
Child caregiving responsibility	
Not caring for a child under the age of 18 *n* (%)	154 (70.3)
**Care Recipients**	
Comorbidity status	
Yes, has at least one comorbidity *n* (%)	147 (67.1)
Living situation	
Lives with caregiver *n* (%)	60 (27.4)

**Table 2:** Patients’ Informal and Formal Care Utilization at T0 and T1 (*n* = 219).

Patients’ Weekly Utilization *Hours*							
		T0 (*SD*)	T1 (*SD*)	Δ (*SD*)	Wilcoxon Signed-Rank Test *z Score*	*n Care Recipients* (%)	
Total informal care		41.9 (76.8)	41.3 (77.0)	−0.6 (4.7)	−1.8	Less care	23 (10.5)
						No change	138 (63.0)
						More care	58 (26.5)
	Support with household tasks	10.7 (22.1)	10.5 (22.0)	−0.2 (1.6)	−1.2	Less care	16 (7.3)
						No change	193 (88.1)
						More care	10 (4.6)
	Support with self-care tasks	11.0 (24.1)	11.0 (24.1)	0.1 (1.4)	0.0	Less care	9 (4.1)
						No change	201 (91.8)
						More care	9 (4.1)
	Practical support	8.8 (16.1)	8.5 (16.0)	−0.2 (1.7)	−1.9 *	Less care	19 (8.7)
						No change	191 (87.2)
						More care	9 (4.1)
	Emotional support	11.5 (25.2)	11.2 (25.4)	−0.2 (3.0)	−2.0 *	Less care	22 (10.1)
						No change	186 (84.9)
						More care	11 (5.0)
Total formal care		n.a.	n.a.	n.a.	n.a.	Less care	49 (22.4)
						No change	135 (61.6)
						More care	35 (16.0)

*Notes*. * *p* < 0.05; n.a. = not assessed; Δ = difference between T0 and T1.

**Table 3:** Informal Caregiver Outcomes at T0 and T1 (*n* = 219).

Informal Caregiver Outcomes *Mean (SD)*				Paired *t*-Test *t*
	T0	T1	Δ	
Subjective burden ^a^	5.1 (2.4)	5.5 (2.4)	0.4 (1.7)	3.2 **
Happiness ^b^	7.0 (1.9)	6.4 (1.9)	−0.6 (1.5)	−6.2 ***
Psychological wellbeing ^c^	7.3 (1.8)	7.2 (1.8)	−0.1 (1.3)	−1.6

*Notes.*^a^ Scale ranges from 0 to 10, with 0 being “not straining at all” and 10 “much too straining;” ^b^ Scale ranges from 0 to 10, with 0 being “completely unhappy” and 10 “completely happy;” ^c^ Scale ranges from 0 to 10, with 0 being “worst imaginable psychological wellbeing” and 10 “best imaginable psychological wellbeing”. **p < 0.01; *** *p* < 0.001; Δ = difference between T0 and T1.

**Table 4:** Impact of Shifts in Care Recipients’ Formal and Informal Care Utilization on Informal Caregivers’ Subjective Burden, Happiness, and Psychological Wellbeing Within Categories of Caregivers (*n* = 219).

	Subjective Burden ^a^				Happiness ^b^				Psychological Wellbeing ^c^			
	T0 (*SD*)	T1 (*SD*)	Δ (*SD*)	Paired *t*-Test *t*	T0 (*SD*)	T1 (*SD*)	Δ (*SD*)	Paired *t*-Test *t*	T0 (*SD*)	T1 (*SD*)	Δ (*SD*)	Paired *t*-Test *t*
No change in formal care hours, Decrease in informal care hours (*n* = 11)	4.9 (2.2)	5.8 (3.0)	0.9 (1.8)	1.7	6.0 (2.9)	5.2 (1.9)	−0.8 (2.4)	−1.1	6.5 (2.3)	6.2 (2.5)	−0.4 (0.9)	−1.3
Increase in formal care hours, Decrease in informal care hours (*n* = 4)	3.5 (3.7)	4.5 (3.0)	1.0 (1.8)	1.1	8.8 (0.5)	6.3 (1.7)	−2.5 (1.7)	−2.9	7.5 (1.3)	7.5 (1.0)	0.0 (0.8)	0.0
Decrease in formal care hours, Decrease in informal care hours (*n* = 8)	4.3 (2.0)	4.8 (2.0)	0.5 (1.6)	0.9	7.1 (1.7)	6.1 (2.1)	−1.0 (0.8)	−3.7 **	7.9 (1.6)	7.1 (2.6)	−0.8 (1.2)	−1.8
No change in formal care hours, No change in informal care hours (*n* = 93)	5.1 (2.4)	5.4 (2.4)	0.3 (1.5)	1.7	7.0 (1.9)	6.6 (1.8)	−0.4 (1.4)	−2.6 *	7.3 (1.9)	7.3 (1.9)	0.0 (1.1)	0.3
Increase in formal care hours, No change in informal care hours (*n* = 16)	4.5 (2.0)	4.6 (2.1)	0.1 (1.0)	0.5	7.3 (1.6)	7.2 (1.7)	−0.1 (0.7)	−0.7	7.4 (1.3)	7.4 (1.4)	0.0 (0.6)	0.0
Decrease in formal care hours, No change in informal care hours (*n* = 29)	5.7 (2.5)	6.6 (2.5)	0.9 (2.0)	2.5 *	6.8 (2.4)	5.5 (2.5)	−1.3 (1.6)	−4.3 ***	7.2 (1.7)	6.8 (1.9)	−0.4 (1.2)	−1.9
No change in formal care hours, Increase in informal care hours (*n* = 31)	5.5 (2.1)	5.5 (2.3)	−0.1 (1.7)	−0.3	7.1 (1.4)	6.6 (1.6)	−0.5 (1.3)	−2.3 *	7.5 (1.8)	7.3 (1.4)	−0.2 (2.0)	−0.5
Increase in formal care hours, Increase in informal care hours (*n*= 15)	4.9 (2.1)	6.2 (2.3)	1.3 (1.5)	3.2 **	6.9 (2.0)	5.7 (1.9)	−1.3 (1.8)	−2.7 *	7.5 (1.6)	7.1 (1.8)	−0.4 (1.1)	−1.5
Decrease in formal care hours, Increase in informal care hours (*n*= 12)	5.3 (2.6)	4.6 (2.6)	−0.7 (1.9)	−1.2	6.8 (1.6)	6.8 (1.8)	−0.1 (1.2)	−0.2	6.9 (1.9)	7.0 (1.7)	0.1 (1.4)	0.2

*Notes.*^a^ Score ranged from 0 to 10, with 0 being “not straining at all” and 10 “much too straining;” ^b^ Score ranged from 0 to 10, with 0 being “completely unhappy” and 10 “completely happy;” ^c^ Score ranged from 0 to 10, with 0 being “worst imaginable psychological well-being” and 10 “best imaginable psychological well-being;”. *p < 0.05; **p < 0.01; ***p < 0.001; Δ = difference between T0 and T1.

## Data Availability

The instruments, data, and methods used are presented in the paper to make it possible for other researchers to replicate the work with their own data. The data will not be made publicly available but will be made accessible after assessment of individual requests.

## Supplementary Materials

The following supporting information can be downloaded at: https://www.mdpi.com/article/10.3390/ijerph192416425/s1, Table S1: Model coefficients for mediation analysis—change in subjective burden; Table S2: Model coefficients for mediation analysis—change in happiness; Table S3: Model coefficients for mediation analysis—change in psychological wellbeing.
