# Supplementary material for: How Do Shifts in Patients with Mental Health Problems’ Formal and Informal Care Utilization Affect Informal Caregivers?: A COVID-19 Case Study
Source: Int J Environ Res Public Health. 2022 Dec 7;19(24):16425. doi: 10.3390/ijerph192416425 (PMC9778175; doi:10.3390/ijerph192416425)
Supplement: Supplementary file 1 [file ijerph-19-16425-s001.zip › ijerph-1995424-supplementary.pdf]

International Journal of
Environmental Research and Public Health

MDPI

Supplementary Materials

How Do Shifts in Patients with Mental Health Problems' Formal and Informal Care Utilization Affect Informal Caregivers?: A COVID-19 Case Study

Supplementary File: Detailed Results of Mediation Analyses

Dependent Variable: Change in Subjective Burden

Table S1. Model Coefficients for Mediation Analysis- Change in Subjective Burden.

			Total Effect Y (Δ Subjective Burden)			M (Δ Informal Care Utilization)			Y (Δ Subjective Burden)	
			Coeff.	SE		Coeff.	SE		Coeff.	SE
Antecedent										
X (Δ Formal care utilization	*c*	X1	0.423	0.322	*a*	-1.953*	0.887	*c′*	0.367	0.326
		X2	0.119	0.290		0.027	0.798		0.120	0.290
M (Δ Informal care utilization)			-	-		-	-	*b*	-0.028	0.025
Covariates										
Overall COVID-19 related concerns			0.137	0.189		-0.418	0.521		0.125	0.190
Age			0.014	0.008		-0.031	0.022		0.013	0.008
Care recipient comorbidity status			0.078	0.254		1.254	0.698		0.113	0.255
Child caregiving responsibility			0.232	0.277		-0.892	0.763		0.207	0.278
Gender			0.687**	0.234		-0.611	0.644		0.670**	0.234
Employment status			0.107	0.259		-0.714	0.713		0.087	0.259
Care recipient living situation			-0.207	0.268		0.613	0.738		-0.189	0.268
Constant	i_1		-1.160	0.634	i_2	1.792	1.744	i_3	-1.109	0.635
			$R^2 = 0.0675$			$R^2 = 0.064$			$R^2 = 0.073$	
			$F(9, 204) = 1.641$, $p = 0.1056$			$F(9, 204) = 1.537$, $p = 0.137$			$F(10, 203) = 1.604$, $p = 0.108$	

Notes. * $p < 0.05$; ** $p < 0.01$; *** $p < 0.001$; coeff. coefficient; F F value; SE standard error.

Dependent Variable: Change in Happiness

Table S2. Model Coefficients for Mediation Analysis- Change in Happiness.

			**Total Effect Y (Δ Happiness)**			**M (Δ Informal Care Utilization)**			**Y (Δ Happiness)**	
			**Coeff.**	**SE**		**Coeff.**	**SE**		**Coeff.**	**SE**
Antecedent										
X (Δ Formal care utilization	*c*	X1	-0.227	0.286	*a*	-1.953*	0.887	*c′*	-0.292	0.288
		X2	-0.294	0.257		0.027	0.798		-0.293	0.256
M (Δ Informal care utilization)			-	-		-	-	*b*	-0.033	0.023
Covariates										
Overall COVID-19 related concerns			-0.449**	0.168		-0.418	0.521		-0.463**	0.168
Age			0.008	0.007		-0.032	0.022		0.007	0.007
Care recipient comorbidity status			-0.121	0.225		1.254	0.698		-0.079	0.226
Child caregiving responsibility			0.626*	0.246		-0.892	0.763		0.600*	0.246
Gender			-0.0840	0.208		-0.611	0.644		-0.104	0.207
Employment status			-0.137	0.230		-0.714	0.713		-0.161	0.230
Care recipient living situation			-0.321	0.238		0.613	0.738		-0.300	0.237
Constant	i_1		-0.100	0.562	i_2	1.792	1.744	i_3	-0.040	0.562
			$R^2 = 0.093$			$R^2 = 0.064$			$R^2 = 0.103$	
			F(9, 204) = 2.324, p = 0.017			F(9, 204) = 1.537, p = 0.137			F(10, 203) = 2.323, p = 0.013	

Notes. * $p < 0.05$; ** $p < 0.01$; *** $p < 0.001$; coeff. coefficient; F F value; SE standard error.

Dependent Variable: Change in Psychological Wellbeing

Table S3. Model Coefficients for Mediation Analysis- Change in Psychological Wellbeing.

			**Total Effect Y (Δ Psychological Wellbeing)**			**M (Δ Informal Care Utilization)**			**Y (Δ Psychological Wellbeing)**	
			**Coeff.**	**SE**		**Coeff.**	**SE**		**Coeff.**	**SE**
Antecedent										
X (Δ Formal care utilization	*c*	X1	-0.007	0.244	*a*	-1.953*	0.887	*c′*	0.019	0.248
		X2	-0.151	0.220		0.027	0.798		-0.152	0.220
M (Δ Informal care utilization)			-	-		-	-	*b*	0.013	0.019
Covariates										
Overall COVID-19 related concerns			-0.172	0.144		-0.418	0.521		-0.166	0.144
Age			-0.009	0.006		-0.031	0.022		-0.008	0.006
Care recipient comorbidity status			-0.197	0.192		1.254	0.698		-0.213	0.194
Child caregiving responsibility			0.100	0.210		-0.892	0.763		0.112	0.211
Gender			-0.400*	0.177		-0.611	0.644		-0.391*	0.178
Employment status			-0.157	0.196		-0.714	0.713		-0.148	0.197
Care recipient living situation			-0.409*	0.203		0.613	0.738		-0.148*	0.197
Constant	i_1		1.146*	0.480	i_2	1.792	1.744	i_3	1.123*	0.482
			$R^2 = 0.064$			$R^2 = 0.064$			$R^2 = 0.067$	
			F(9, 204) = 1.558, p = 0.130			F(9, 204) = 1.537, p = 0.137			F(10, 203) = 1.445, p = 0.163	

Notes. * $p < 0.05$; ** $p < 0.01$; *** $p < 0.001$; coeff. coefficient; F F value; SE standard error.
